# A mentorship and incubation program using project-based learning to build a professional bioinformatics pipeline in Kenya

**DOI:** 10.1371/journal.pcbi.1010904

**Published:** 2023-03-02

**Authors:** Ruth Nanjala, Festus Nyasimi, Daniel Masiga, Caleb Kipkurui Kibet

**Affiliations:** 1 International Centre of Insect Physiology and Ecology, Nairobi, Kenya; 2 Kennedy Institute for Rheumatology, Nuffield Department of Orthopaedics, Rheumatology and Musculoskeletal Sciences, University of Oxford, United Kingdom; 3 The University of Chicago, Chicago, Illinois, United States of America; McGill University, CANADA

## Abstract

The demand for well-trained bioinformaticians to support genomics research continues to rise. Unfortunately, undergraduate training in Kenya does not prepare students for specialization in bioinformatics. Graduates are often unaware of the career opportunities in bioinformatics, and those who are may lack mentors to help them choose a specialization. The Bioinformatics Mentorship and Incubation Program seeks to bridge the gap by laying the foundation for a bioinformatics training pipeline using project-based learning. The program selects six participants through an intensive open recruitment exercise for highly competitive students to join the program for four months. The six interns undergo intensive training within the first one and a half months before being assigned to mini-projects. We track the progress of the interns weekly through code review sessions and a final presentation at the end of the four months. We have trained five cohorts, most of whom have secured master’s scholarships within and outside the country and job opportunities. We demonstrate the benefit of structured mentorship using project-based learning in filling the training gap after undergraduate programs to generate well-trained bioinformaticians who are competitive in graduate programs and bioinformatics jobs.

This is a *PLOS Computational Biology* Methods paper.

## Introduction

The field of bioinformatics has grown substantially since the completion of the first draft of the human genome in the 1990s [1]. The tremendous increase in the volume of data generated from sequencing has increased the demand for bioinformaticians who can skillfully interpret large and complex datasets [2]. Although bioinformatics is increasingly crucial for life sciences research, undergraduate biology education is not structured adequately to incorporate bioinformatics skills and knowledge. Curriculum gaps can limit biology students from reaching their full educational potential, restricts their job options, and hinders research progress [3].

The countries of the global north have incorporated the core competencies of bioinformatics into undergraduate life sciences courses [3], including fully approved graduate degree training programs in bioinformatics. Therefore, the transition to research and graduate school is seamless, as students already have basic research and computational biology skills. Short bioinformatics courses that last only a few days to a few weeks fill the skill gap in resource-constrained settings. The introductory [4] and intermediate [5] bioinformatics training of Human Heredity and Health in Africa Bioinformatics Network (H3ABioNet) [6] has successfully used African bioinformatics experts to reach large cohorts of trainees through blended learning. However, these courses lack structured mentorship and project-based learning critical for skills retention and bioinformatics career specialization [2,7].

Africa, regarded as the cradle of humankind [8], has a high genetic diversity and a high burden of infectious diseases. Bioinformatics capacity is required to improve research in Africa and address the burden of disease [9]. Although advanced bioinformatics training remains a privilege for countries with cutting-edge scientific resources [10], some African countries, South Africa [11], Mali [12], Nigeria [13], Ghana [10], and Uganda [7] have worked to close such gaps through different bioinformatics capacity-building programs. In Kenya, there is no structured bioinformatics mentorship.

Few organizations in Kenya—International Centre of Insect Physiology and Ecology (*icipe*), the International Livestock Research Institute (ILRI), Kenya Medical Research Institute, Wellcome Trust (KEMRI-WT)—play a crucial role in closing this gap through short-term training and workshops, but this is not enough. In the absence of ongoing support, those who receive training cannot continue learning and utilize the skills gained through research projects. Furthermore, short-term training is sustainable because trainees do not retain their bioinformatics skills.

The Human Hereditary and Health in Africa (H3Africa) project led to the demand for bioinformaticians who could analyze genomic data generated from H3Africa projects [14]. The Fogarty International Center, which supports H3Africa projects, supported bioinformatics capacity development by sponsoring master’s and PhD students through projects such as the Eastern Africa Network for Bioinformatics Training program (EANBiT) [15] and Nurturing Genomics and Bioinformatics Capacity in Africa [16]. However, there remains a gap in how to attract highly motivated graduates interested in pursuing bioinformatics as a career. Short-term training fills additional gaps in niche areas such as metagenomics, but structured project-based immersive training and mentoring offer the best impact in establishing sustained interest [17].

## Bioinformatics Incubation and Mentorship Program Design

We established the Bioinformatics Incubation and Mentorship Program to fill the gap by creating a foundation for bioinformatics career development. The program recruits six highly motivated students interested in bioinformatics every four months to *participate in* structured training and mentorship. The aim is to generate a pool of competitive bioinformatics trainees who can proceed to master’s degree training or support ongoing research.

The mentorship program is based on a project-based learning approach to increase bioinformatics capacity. We modeled the incubation program to impart key bioinformatics competencies to bioinformatics scientists, according to ISCB [18], with clear learning objectives and results. The program aims to provide knowledge and skills in bioinformatics competencies: data management; bioinformatics tools, resources, and their use; the scientific discovery process and the role of bioinformatics in it; computer science systems basics; scripting and programming, open source, and version control tools, all of which are relevant to the bioinformatics discipline.

### Project-based learning approach

The selected interns undergo rigorous training within the first one and a half months. The training covered by different tutors is meant to introduce the field of bioinformatics, guide the interns in designing their purpose road maps, teach key people skills, and introduce them to data management, open science, and reproducible research (Table 1). In addition to bioinformatics skills, the program includes information literacy workshops on how to access subscribed electronic resources, publish in credible journals, maintain a scholarly presence online, and manage references.

**Table 1:** Bioinformatics Incubation Training Program. The topics covered in each module, and the resources used for the training are available in the GitHub repo: https://github.com/mbbu/training-materials-and-resources.

Time period	Course structure	ISCB core competencies	Blooms taxonomy
Week 1	Onboarding and general overview of the Molecular Biology and Bioinformatics Unit	O–Effective teamwork to accomplish a common scientific goal.	Knowledge
	Introduction to collaborative documents–Slack, HackMD	I–GUI/ Web-based computing skills appropriate to the discipline	application
	Developing the purpose roadmap, objective, and goals	P–Engage in continuing professional development in bioinformatics	evaluation
	Open Science and Data Management, and Reproducibility (introductory lecture on open science, FAIR data, and data management plans)	P–Engage in continuing professional development in bioinformatics	application
Week 2	Version Control with Git and GitHub	J–Command line and scripting-based computing skills appropriate to the discipline.	application
		I–GUI/Web-based computing skills appropriate to the discipline	application
	Introduction to Unix Shell–BASH scripting	J–Command line and scripting-based computing skills appropriate to the discipline.	application
	Brief introduction of the bioinformatics field	F–Bioinformatics tools and their usage.	evaluation
	Introduction to Next-Generation Sequencing and file formats	C–Biological data generation technologies	Knowledge to analysis
	Soft skills (Personality, Motivation, Leadership attributes and styles)	N–Effective communication of bioinformatics and genomics topic with a wide range of audiences	application
Week 3	Python programming (basic data types and operations, string manipulation, data structures: lists, tuples, sets and dictionaries, control statements, functions, scripting with python)	J–Command line and scripting-based computing skills appropriate to the discipline.	analysis
	Soft skills (Communication and presentation skills, giving feedback and Conflict resolution)	N–Effective communication of bioinformatics and genomics topic with a wide range of audiences.	application
Week 4	R Programming language (Introduction to R and RStudio, data structures in R, exploring data frames, subsetting data)	J–Command line and scripting-based computing skills appropriate to the discipline	analysis
		E–Statistical research methods in the context of molecular biology, genomics, medical, and population genetics research	application
	Introduction to phylogenetics	B–Depth in at least one area of biology	application
	Workflow languages (Nextflow and Snakemake) and Containerization (Docker and Singularity)	F–Bioinformatics tools and resources and their usage	application
	Soft skills (Career planning and peer mentoring)	N–Effective communication of bioinformatics and genomics topics with a wide range of audiences.	analysis
Week 5	Data Science and Machine learning (Machine learning Concepts, Introduction to Linear Regression, Random Forest and Decision Trees, Feature Engineering in Genomics)	K–Construction of software systems of varying complexity based on design and development principles	knowledge
	Introduction to HPC and Cloud Computing (SSH, moving data, Slurm scheduler, lecture on AWS)	H–Computing requirements appropriate to solve a given scientific problem	application
Week 6 and Week 7	Mini-project on reproducing bioinformatics data analysis from published papers	B–Depth in at least one area of biology	application
		I–GUI/Web-based computing skills appropriate to the discipline	analysis
Week 8	Information literacy workshop (Publishing online, maintaining online scholarly presence, Reference management)	N–Effective communication of bioinformatics and genomics topic with a wide range of audiences.	knowledge
	Molecular Biology Laboratory tour	NA	NA
Week 9–Week 17	Mini-projects	B–Depth in at least one area of biology	application
		O–Effective teamwork to accomplish a common scientific goal	analysis
		F–Bioinformatics tools and resources and their usage	application
		E–Statistical research methods in the context of molecular biology, genomics, medical, and population genetics research	application
	Journal club presentations.	N–Effective communication of bioinformatics and genomics topic with a wide range of audiences.	analysis
		A–General Biology	comprehension

The project-based learning approach is implemented in two phases: reproducing bioinformatics analysis in a published paper and data from ongoing projects at *icipe*. In the last two weeks of the second month, the interns reproduced methods in a paper that would have applied techniques taught in the first one and a half months—sequence analysis, phylogenetics, and NGS—and shared their data. The interns are assessed through a final presentation at the end of the month; the strengths of the intern are evaluated to facilitate appropriate assignments to projects from the second phase. In the second phase, the mini-projects carried out within two months related to ongoing genomics work at *icipe*. In the first week, the interns present how they would tackle the project, how they would collaborate, and the tools they would use. Weekly code review sessions help track the progress of interns and enable code debugging and documentation using GitHub wikis and GitHub projects.

### Methods of program delivery

**Fig 1:**
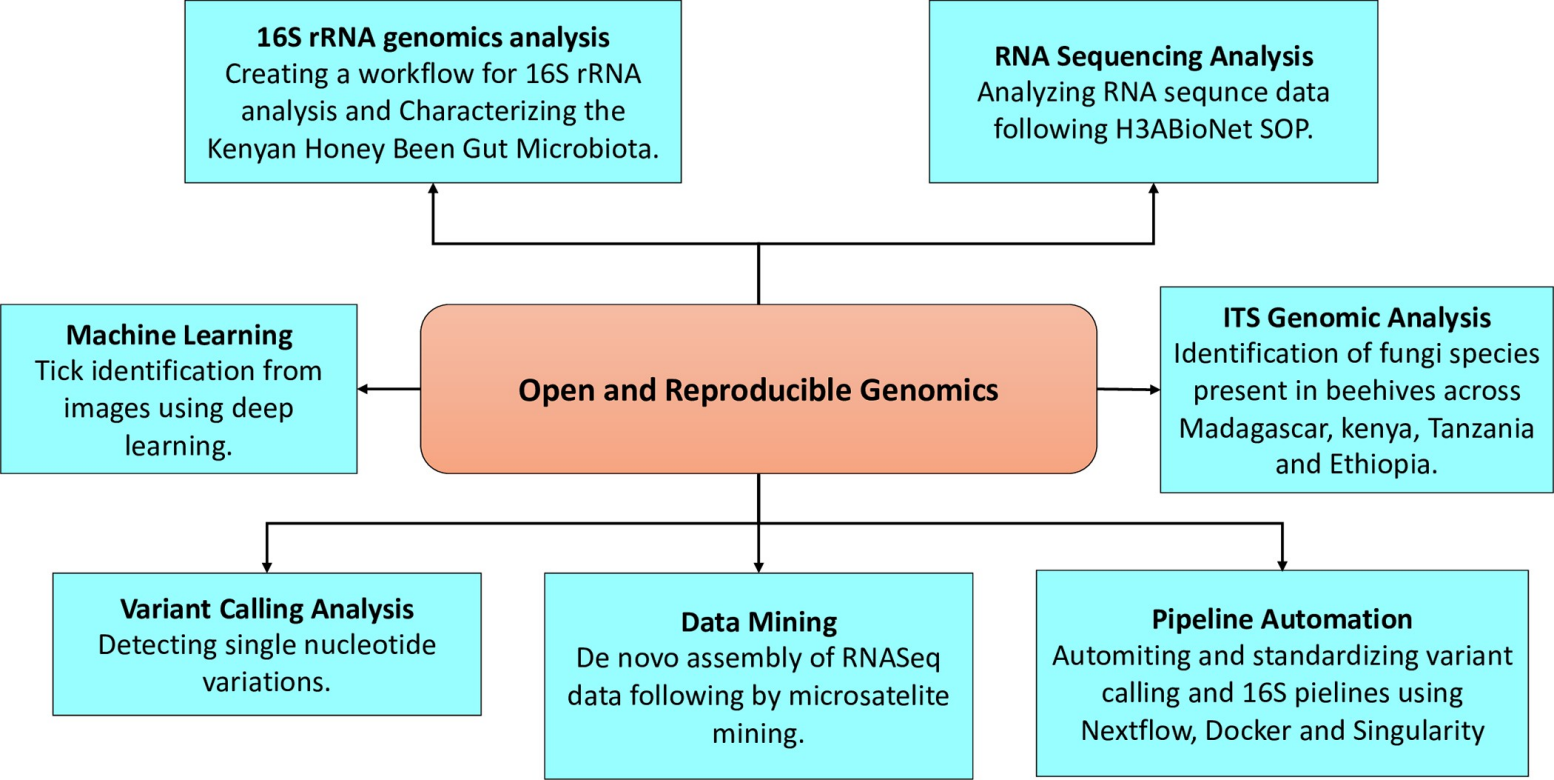
Mini-projects design and sample projects. The project-based learning employed in the incubation program is centered on open and reproducible research.

The various fields of bioinformatics are condensed into a progression of self-contained modules taught in logical order (Fig 1). The curriculum design also included a practical set of abilities that are crucial to putting that information into practice. Before teaching advanced topics, essential topics are taught.

Using seven contact hours per day for six weeks, the course is delivered using both theory sessions in the morning and practical hands-on sessions in the afternoon, which are handled by the local Senior Faculty, the program coordinator, and MSc fellows as teaching assistants. When mentees require them outside those scheduled times, the program coordinator and teaching assistants provide additional practical sessions and office hours. The interns are then tested at the end of each module through regular reading and practical exercises.

Once the key concepts have been delivered in the first six weeks, the interns are put into groups of 2 or 3 based on shared interests in readiness for the mini-projects phase. First, the interns are tasked to reanalyze data from published papers and their results compared with those published study. The interns are then assigned projects from ongoing research at the Centre, where they work closely with the scientists to analyze and interpret the data. During this phase, they receive help from the local senior faculty, program coordinator, and MSc fellows through weekly code review sessions. They then make a final presentation to summarize their mini-projects on the last day of the internship.

In addition to standard bioinformatics topics (sequence analysis, phylogenetics, NGS, and workflows), journal club meetings, soft skills sessions, and the information literacy workshop are part of the internship program. These sessions are included because they are crucial to the professional development of the mentees. We also guide interns in job and MSc applications through mock reviews and interviews in the last month. The cover letters, CVs, and motivations of the fellows are reviewed, and feedback is provided based on the skills gained by the interns.

Mini-projects focused on open and reproducible genomic analysis (Fig 1).

### Recruitment

The Bioinformatics Incubation and Mentorship Program is designed for highly motivated students interested in bioinformatics recruited through widely advertised calls and direct recommendations from relevant university departments. It is hosted at the *icipe* Molecular Biology and Bioinformatics Unit (MBBU). To be selected for the program, interested students from different universities in Kenya submit applications to *icipe* through a web-accessible form developed in REDCap [19,20]. The application includes a motivation letter and a curriculum vitae detailing their background.

For each round, we shortlist at least 15 applications for an interview. The applications are critically reviewed through an initial long list to ensure completeness and essential qualifications. We are interested in highly motivated students who have completed their undergraduate degrees. The desire to pursue Bioinformatics should not be an afterthought and must have put some effort into writing their motivation letter with clarity on how it fits their career plan. At least two reviewers then score long-listed applications based on whether the applicant is motivated to pursue a career in bioinformatics, the clarity of how the internship fits their career plan, and the quality of the application.

**Fig 2:**
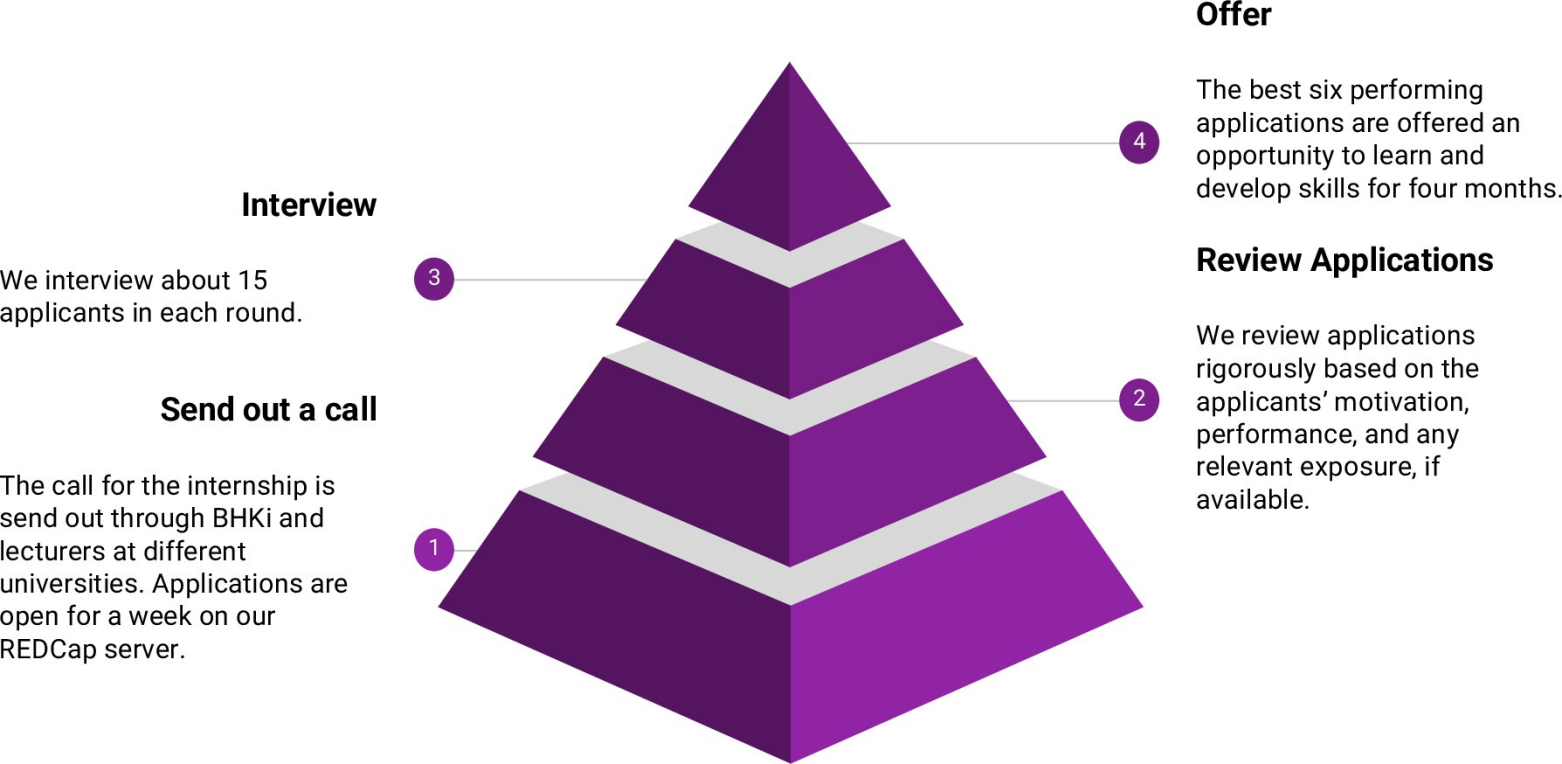
Recruitment process.

The rigorous selection process is vital for success, as we work with highly motivated and curious students. Interview questions aim to reveal what makes the applicant uniquely qualified for the internship based on prior interests, problem-solving skills, curiosity, and how the training aligns with their career goals. The questions were as follows: (1) For this internship, tell us who you are and what makes you uniquely qualified for the position. (2) You are faced with a challenge you have not encountered before; briefly tell us about your thought process and the approach to solving it. (3) What are your expectations from the internship and how does it align with your goals? Three interviewers select six interns to join the program through independent ranking. A summary of the recruitment process is described in Fig 2.

## Impact of the program

The internship program has been run successfully for five iterations since October 2020, mentoring 27 students. The program has generated a pool of competitive students who have secured master’s degrees within and outside the country and job opportunities. Some interns continued various ongoing projects at *icipe* and with our collaborators. Table 2 documents the post-internship positions secured by some of the interns. Those not included [10] are exploring their next steps in the bioinformatics field and as teaching assistant positions for H3ABioNet courses and the Carpentry organization, while others have taken short courses and mini-projects to perfect their bioinformatics skills.

**Table 2:** Post-internship progression.

Intern	Cohort	Background	Internship period	Student’s current position
Intern 1	1	BSc Molecular Biology	October 2020–January 2021	MSc Bioinformatics candidate, Kenya
Intern 2	1	BSc Microbiology and Biotechnology	October 2020–January 2021	Software Engineer, Kenya
Intern 3	1	BSc Biotechnology	October 2020–January 2021	MSc Bioinformatics (the Netherlands, Europe)
Intern 4	1	BSc Microbiology	October 2020–January 2021	MSc Genetics (Brazil, South America)
Intern 5	2	BSc Molecular Biology	February 2021–May 2021	MSc Bioinformatics candidate, Kenya
Intern 6	2	BSc Applied Bioengineering	February 2021–May 2021	MSc Bioinformatics candidate, Kenya
Intern 7	2	BSc Medical Biology and Chemistry	February 2021–May 2021	Assistant Research Officer, Research Organization, Kenya
Intern 8	2	BSc Biochemistry and Molecular Biology	February 2021–May 2021	Masters in Life Sciences and Health (Europe)
Intern 9	2	BSc Marine Biology	February 2021–May 2021	MSc Life Science (Belgium, Europe)
Intern 10	3	BSc Botany	June 2021–September 2021	Scientist at a Pharmaceutical company
Intern 12	3	BSc Biochemistry	June 2021–September 2021	Bioinformatician, Research Organization, Kenya
Intern 13	3	BSc Biochemistry	June 2021–September 2021	MSc. Bioinformatics candidate, Kenya
Intern 14	4	BSc Statistics	October 2021–January 2022	Intern, Industry, Kenya
Intern 15	4	BSc Medical Biochemistry	October 2021–January 2022	Software developer, Industry, Kenya
Intern 16	4	BSc Genomic Sciences	October 2021–January 2022	Intern, Research Organization, Kenya
Intern 17	5	BSc Biochemistry and Molecular Biology	February 2022–May 2022	Research Assistant, Bioinformatics, Germany

### Feedback from the participants

We assess and monitor the progress and impact of the bioinformatics mentorship and incubation program through pre- and post-internship surveys, weekly updates, and code reviews. The interns join the program with the expectations of learning bioinformatics in depth, improving presentation skills, learning to code, creating meaningful friendships and networks, being mentored, interacting with leading scientists, and gaining in-depth knowledge of genomics and genetics. Before the program, the research interests of the interns were more generalized, but after the internship was completed, the interests aligned. Some of the examples are as follows:

“Research has always been my interest, and training has shaped me in an interesting field of metagenomics. An added value was that I got to do metagenomics analysis as a mini-project. Yes, my research interests have changed.”

“Before the internship, I wasn’t quite sure what I wanted to venture into, but now I am interested in using bioinformatics tools to study infectious and non-infectious diseases.”

“Before training, I wanted to be a genomics researcher. Going out, I am still interested in genomics research, but the internship program opened my mind to find a field to specialize in, and that’s what I’m still trying to figure out.”

**Fig 3:**
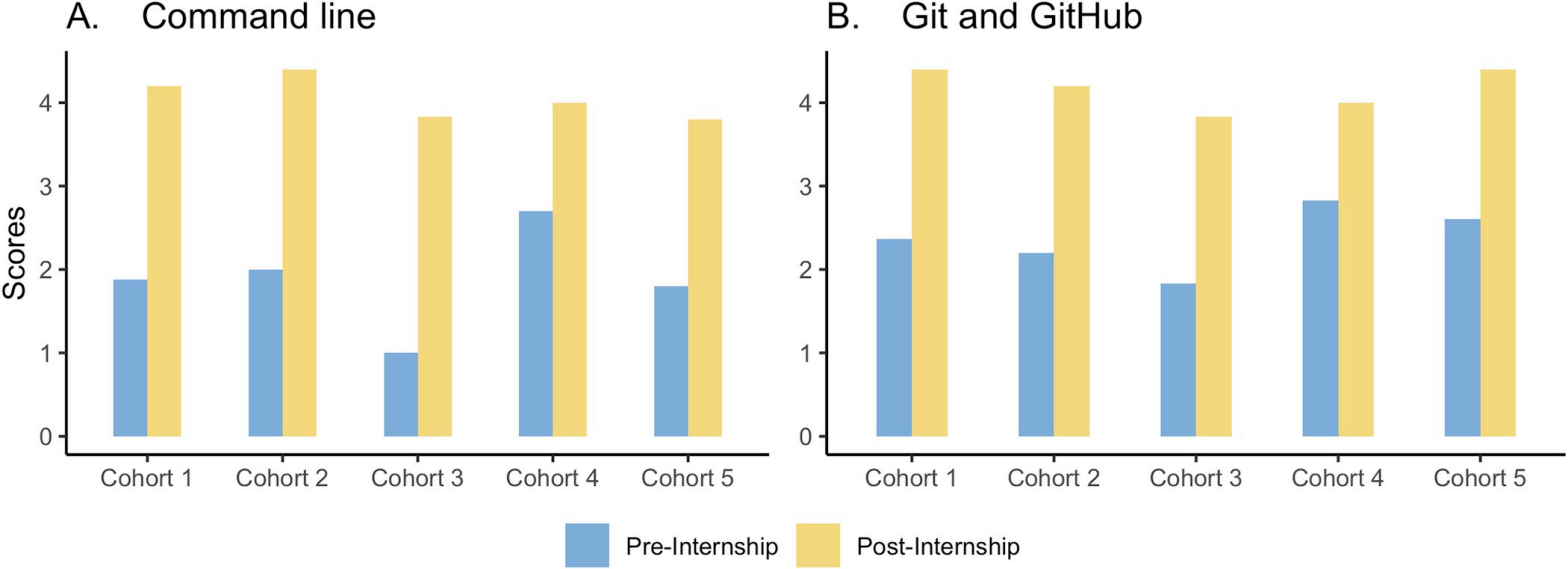
The figures summarize the interns’ competencies *in Command line and Git* acquired from the modules pre- and post-internship. Scores: 1 –Never used, 5 –Advanced.

**Fig 4:**
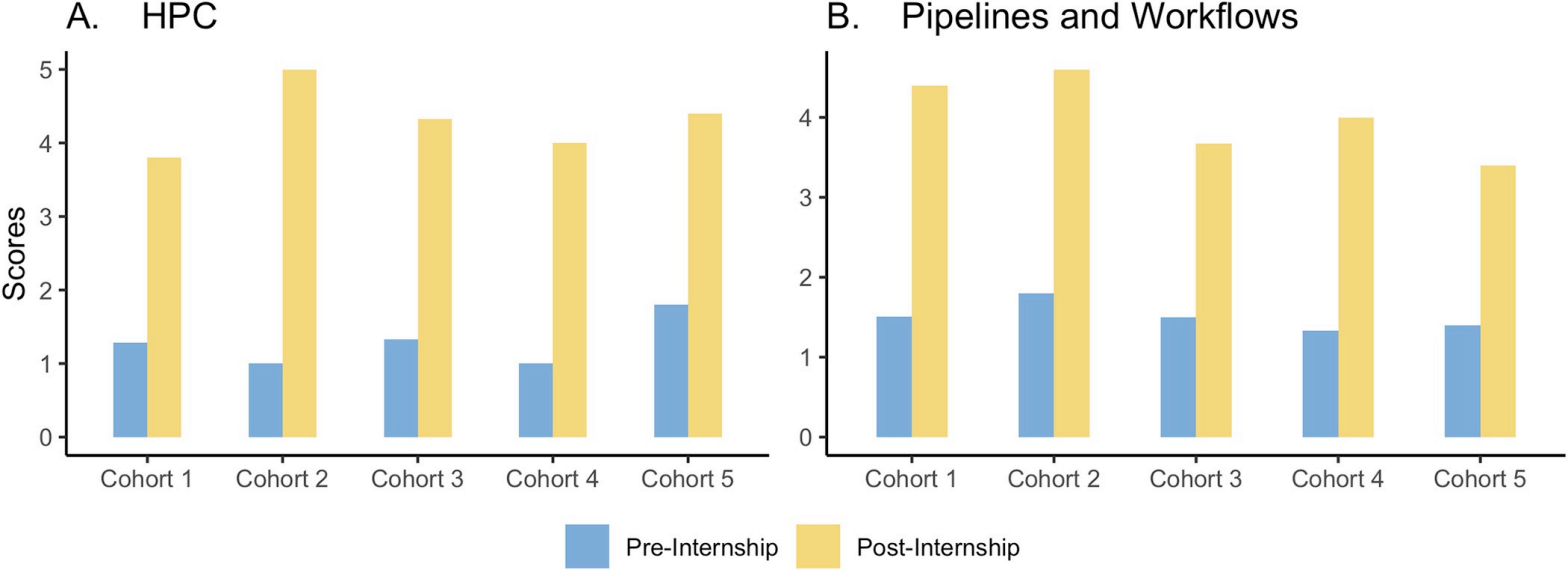
The figures summarize the interns’ competencies *in HPC*, *and Pipelines* acquired from the modules pre- and post-internship. Scores: 1 –Never used, 5 –Advanced.

**Fig 5:**
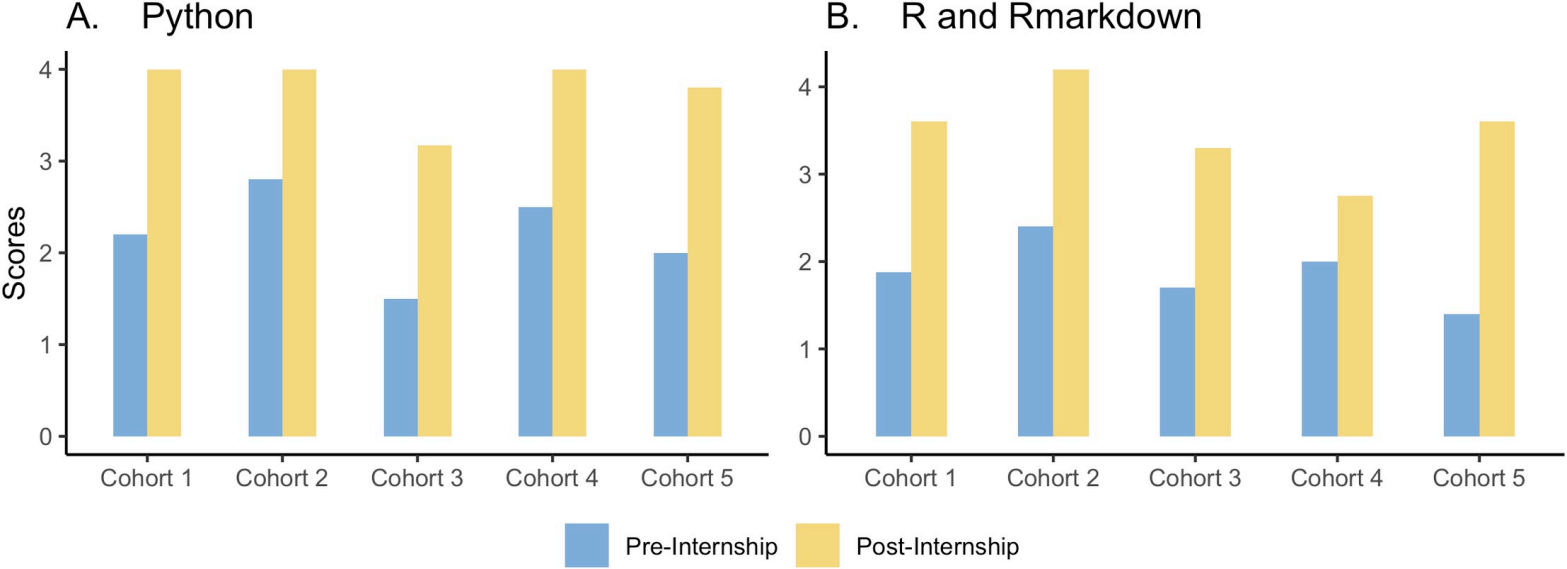
The figures summarize the competencies of the interns *in Python and R* acquired from the modules pre- and post-internship. Scores: 1 –Never used, 5 –Advanced.

The interns improved their competencies in command line (Fig 3A), Git and GitHub (Fig 3B), working with the HPC (Fig 4A), building bioinformatics pipelines and workflows (Fig 4B), Python (Fig 5A), and R and Rmarkdown (Fig 5B).

**Fig 6:**
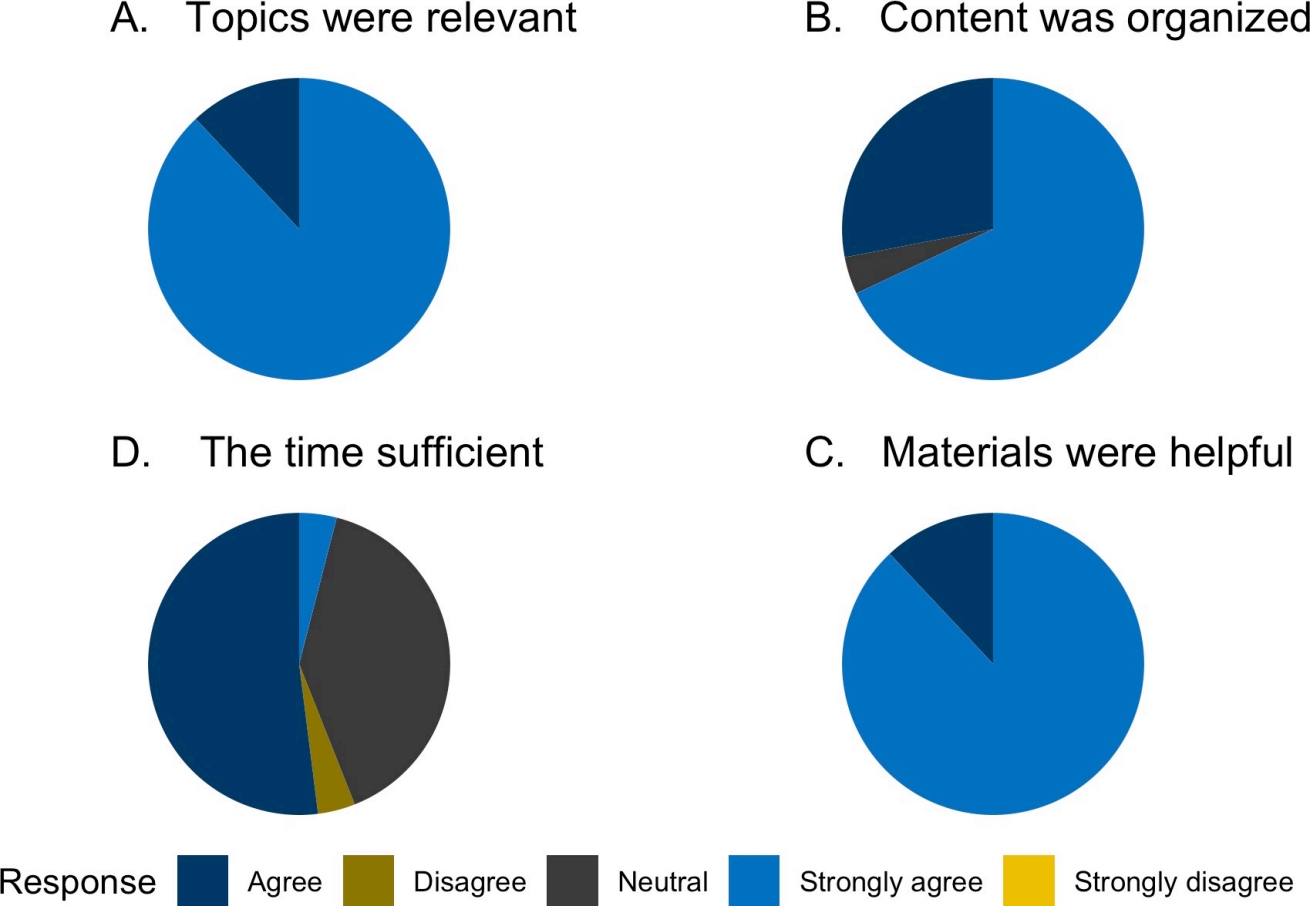
A summary of the responses on the relevance of the topics covered content organization, material distribution, and time allocation.

As part of the post-internship survey, the interns highlighted their training experience, as shown in Fig 6. They noted that the topics, content, and materials were helpful and sufficient but highlighted that the time was not always enough.

In addition to quantitative feedback, the interns reflected on their experience through a blog post published on our program page.

We highlight one representative example in the following:

“Coding has always fascinated me. Being from an applied bioengineering (genomics-based) background, I actively sought a bridge between the two. The internship allowed me to learn and polish my programming skills under the guidance of a tutor and to see its application in multi-omics data processing of real-life problems. In addition, it has cultivated a self-learning discipline that has played a key role in solidifying and expanding my bioinformatics knowledge. The skills and experience gained during the internship gave me a competitive advantage when applying for bioinformatics opportunities, paving the way to obtain the EANBiT Master’s fellowship. The internship increased my resilience and self-discipline, which still plays a key role in my MSc Bioinformatics journey.”

### Successes, challenges, and future improvements

#### Key achievements

Design and implementation of a project-based learning bioinformatics training curriculum. Using the curriculum, we have mentored and trained 27 interns, generating a pool of fellows to join the bioinformatics training pipeline and contribute to the bioinformatics capacity in the continent.

#### Reproducibility

A key component of the mentorship program is to enhance collaboration and reproducible research. All mini-projects have been properly documented on the organization’s GitHub account, enhancing reproducibility. The curriculum and the resources used for the internship program are publicly available on the MBBU GitHub pages and can be used to establish a similar program.

#### Sustainability through the Mentorship Series

To continue mentoring interns beyond the internship period, we launched the Science Journey Seminar series, where interns interact with scientists worldwide through seminars. These seminars highlight paths and journeys of established and upcoming scientists to motivate, enlighten, and guide them on potential career paths in bioinformatics.

#### Challenges

The fast pace and turnaround of the program meant that we had to continuously design projects that fit the interest of the interns. Sometimes, however, some interns’ interest did not align with the work at the Centre, or that data-rich projects requiring their expertise were not readily available from within the host institute. We addressed this by forging collaboration outside the institute to ensure that the interest of fellows was catered to. The short duration of the mentorship program, though by design, may not allow the completion of some of the projects. In these cases, we have had fellows retained by the projects after the internship.

## Discussion and conclusions

A substantial increase in genomics and proteomics data has catalyzed the demand for well-trained bioinformaticians [21], but undergraduate courses do not prepare students for specialization in bioinformatics, and training opportunities are insufficient [22]. We established a structured project-based bioinformatics incubation and mentorship program to prepare undergraduate students for a career in bioinformatics. The entire program is a mentoring and incubation program rather than a typical internship program. Interns are taught, supervised, and mentored by researchers and MSc in bioinformatics students in the Molecular Biology and Bioinformatics Unit at *icipe*. Mentorship is vital as the research mentor influences the professional growth of the intern [23]. The curriculum covers key bioinformatics competencies and critical soft skills that culminate in mini-projects tailored to meet the interests of the interns and facilitate the retention of skills. By reproducing research articles after structured training, interns can grasp how experts have applied the skills and tools they have learned. Together with the presentations at the journal club, they can critically improve their ability to judge the validity and rigor of scientific approaches and findings [24] and be exposed to the rapidly changing literature [25]. Reproducing research articles is thus an essential component of the training as the interns get to understand the benefits practically.

Structured training and dedicating the last two months of the internship program to a mini-project contributed to the success of the program. The projects span various topics based on the interests of the interns, available data, and ongoing research within *icipe*. The emphasis on project-based learning (PBL) is vital in cementing the knowledge and skills learned, an integral approach yet to be widely implemented in bioinformatics curricula [20]. The PBL increases the degree of participation of the trainees [26], as previously demonstrated through a five-week summer school [23]. Students become protagonists in the teaching–learning process and develop a more critical problem solving mindset as we challenge them to apply skills to real problems, attaching purpose and benefit to the skills acquired, thus ensuring retention [27].

During the mini-projects, we train the interns to think critically and computationally, as this helps them understand research problems and code debugging. “Confidence in dealing with complexity, persistence in working with difficult problems, tolerance of ambiguity, the ability to deal with open-ended problems, and the ability to communicate and work with others to achieve a common goal” are all skills that computational thinking helps students develop [28]. The program builds collaboration skills through group tasks and mini-projects and advocates for Open Science and reproducible analysis, demonstrated by documenting their work in public GitHub repositories. Documenting is key as it helps create code that is tidy, error-free, and reusable [29].

Weekly code review sessions facilitate continuous feedback and help uncover bugs and code issues with a fresh set of eyes. They also provide room for new ideas and easier ways to perform tasks. In addition, they help track the progress of a project and identify gaps and ways to improve the project. Bioinformatics research and practice have significant implications for life sciences, and effective quality assurance techniques such as code reviews and testing are vital to ensuring software quality [30]. We advocate for open science during the internship. The collaborative and reproducibility components of Open Science are critical to their development as scientists because they lead to more citations, media attention, possible collaborators, career prospects, and funding opportunities [31].

The interns give feedback occasionally by filling out pre-internship surveys, verbal feedback after every taught module, and post-internship surveys. The pre-internship surveys highlight the training needs of the interns and point out areas that require more emphasis during training. It also helps to highlight the research interests of the interns so that we can help them find suitable mentors. Verbal feedback after every taught module is essential in gauging whether the interns understood the topic of interest. The post-internship survey highlights the success of the internship journey and the areas that need improvement.

Figs 3, 4 and 5 show a tremendous improvement in the bioinformatics capacity of the interns after the internship considering they had a short time to grasp and practice the modules taught. The Bioinformatics Incubation and Mentorship Program has increased the number of well-trained bioinformaticians and helped build the capacity for bioinformatics trainers; some interns have become certified Carpentries Instructors.

We have established a structured project-based mentorship and incubation program that fills the bioinformatics training gap between undergraduate and graduate programs or job opportunities. The program trainees are highly competitive for MSc programs and job placements within and outside Kenya, demonstrating the benefit of mentorship in filling the transition gap, thus creating a pool of highly motivated trainees for recruitment to graduate school, and supporting genomic data analysis in Africa.
